# Curve progression 25 years after bracing for adolescent idiopathic scoliosis: long term comparative results between two matched groups of 18 versus 23 hours daily bracing

**DOI:** 10.1186/s13013-016-0065-z

**Published:** 2016-03-09

**Authors:** Stavros Pellios, Eustathios Kenanidis, Michael Potoupnis, Eleftherios Tsiridis, Fares E. Sayegh, John Kirkos, George A. Kapetanos

**Affiliations:** Academic Orthopaedic Unit, Aristotle University Medical School, General Hospital Papageorgiou, Thessaloniki, Greece

**Keywords:** Adolescent Idiopathic Scoliosis, Boston brace, Part time bracing, Full time bracing, Comparative study, Long term data

## Abstract

**Background:**

Scoliotic curves do not necessarily stop progressing at skeletal maturity. The factors that influence curve behavior following bracing are not fully determined. Our objectives were to evaluate the loss of the scoliotic curve correction in a cohort of patients treated with bracing during adolescence and to compare the outcomes of 18 versus 23 h of bracing at a mean of 25 years post brace removal.

**Methods:**

Seventy-seven patients, who were successfully treated for Adolescent Idiopathic Scoliosis with Βoston brace, were re-evaluated 25 years after the end of their treatment. Patients were further divided in 2 matched groups; those wearing the brace for 23 h and those not wearing the brace at school-time, limiting the application of the brace to 18 h. The mean scoliotic curve was compared between groups before, during, just after bracing and 25 years post bracing. Validated in patients’ native language forms of Short Form 36 and Oswestry Disability Index questionnaires were used to compare the quality of life between groups 25 years post bracing.

**Results:**

The mean age of the cohort was 40.4 (±3.2) years. They underwent long term follow up at a mean of 25.16 (±2.69) years after brace removal. The mean cohort scoliotic curve increased by 3.9 (±6.69) at 25 years since brace removal. There was however no significant difference in the mean Cobb angle of the cohort between pre brace and long term follow up period (*p* = 0.307).

The 18 and 23 h application groups were comparable according to demographics and several bracing and scoliotic curve parameters. There was no significant difference in the mean curve magnitude between 18 and 23 h application groups at brace removal (*p* = 0.512) and at 25 years follow-up (*p* = 0.878). There was also no significant difference in the mean score of Quality of Life questionnaires between groups at long term follow up.

**Conclusion:**

Scoliotic curves do not necessarily stop progressing after bracing. Bracing is effective treatment method with good long term results in appropriate patients. Since compliance was not objectively measured, we don’t feel confident to give any indication about everyday dosage.

## Background

Adolescent Idiopathic Scoliosis is a three dimensional spinal deformity of puberty [1]. It is characterized by a great diversity among children. A small percentage of children have progressive curves requiring treatment [2]. The risk factors that influence curve behavior over time have not been fully determined yet [3].

Rigid thoracolumbar orthoses are the mainstay of conservative treatment of Adolescent Idiopathic Scoliosis [4]. Bracing proved to be better treatment choice compared to observation in patients who are at risk for curve progression [5, 6]. The benefit of bracing is correlated with longer daily application time; compliance however remains an issue [5].

Scoliotic curves do not necessarily stop progressing after bracing and skeletal maturity [7, 8]. Knowledge of the factors that influence curve behavior during or following bracing is crucial for the prognostication of patients with Adolescent Idiopathic Scoliosis. Difficulties in the integration of randomised clinical trials render the long term cohort studies the main source of information for scoliosis [5]. The aim of our study was to present 25 years long term follow up data on 77 patients, further divided in 2 matched groups wearing the Boston brace for 18 or 23 h a day for 3 years for the treatment of Adolescent Idiopathic Scoliosis. Our primary aim was to evaluate scoliosis progression 25 years after removal of the brace in a group of Adolescent Idiopathic Scoliosis patients. Secondarily we also tried to determine whether the time of brace application influenced the long term Boston results.

## Methods

### Patient population

One hundred seventeen patients who were treated for Adolescent Idiopathic Scoliosis with Βoston brace enrolled in a prospective cohort study between 1978–1993 in our Academic Institution. All patients successfully concluded their brace treatment. This cohort was originally evaluated over a period of 8 years namely 3 years of treatment and 5 years of post-bracing follow up. We re-evaluated 77 patients of the same cohort 25 years after the end of their treatment to ask loss of correction and quality of life. The study was approved by the Ethics and Research Committee of our academic institution which is a tertiary referral center for orthopaedics and it was conducted in accordance with the World Medical Association Declaration of Helsinki, 1975, as revised in 1983. Informed consent was obtained by all participants.

### Index diagnostic criteria and treatment methods

Inclusion criterion was adolescent male and female patients over 10 years of age diagnosed with Adolescent Idiopathic Scoliosis. Indication for bracing was recommended for major scoliotic curve ranging between 20–40^0^ and a subsequent progression > 5° following 6 months interval in an immature skeleton (Risser sign < 4). All patients were braced with Boston brace for a mean period of 3 years (ranging between 1 to 8 years). Patients were advised to wear the brace for 23 h per day (Full Time Application). Some of the adolescents however did not wear the brace during school time and lessened application to 18 h per day (Part Time Application).

Prerequisites for bracing discontinuation were skeleton maturation, 3 years pass over menarche for the female patients or no deterioration of scoliotic curve for a period of 24 month follow-up. Skeletal maturity was defined as a Risser sign ≥ 4 or the age of 16 and 18 years for girls and boys respectively [9]. Short term follow-up was discontinued 5 years after brace removal. Radiology reports with measurements of the deformity were available for all patients.

### Reevaluation 25 years after removal of the brace

#### Clinical evaluation

We retrospectively followed up this cohort 25 years later. Out of 117 patients 77 came for a long-term follow-up. Demographics were recorded. They were observed in the standing erect position and during the forward bending test for asymmetries of the lateral contours of the trunk, rib hump asymmetry, shoulders and scapulae elevation.

#### Radiographic evaluation

A full length anteroposterior and lateral radiograph from the base of the skull to the coccyx was taken. The Cobb angle measurement of the major and the minor curve, the type of the scoliotic curve and the apical vertebra were re-evaluated. Curve types were classified according to the Scoliosis Research Society Adult Deformity Classification [10]. Curves measurements were performed by the same physician using the Cobb method and the same end vertebrae before and after brace application. Rotation of the apical vertebra was rated using Perdriolle method [11].

#### Quality of life

Validated in patients ’native language forms of Short Form 36 and Oswestry Disability Index questionnaires were used [12, 13]. The Short Form-36 is a measure of general health status. The Oswestry Disability Index was used to quantify pain related disability due to low back pain.

### Sub group analysis

Seventy-seven patients were divided in 2 groups based on the daily brace application. Those wearing the brace for 23 h and those not wearing the brace at school-time, limiting the application of the brace to 18 h. The group selection was not imposed by the study design but was occurred naturally by the patients willing to wear the brace during school-time or not.

### Statistical analysis

The necessary sample size estimation to provide valid results was decided based on the reported “loss of curve correction” in previous study [8]. Taking into consideration the fact that the reported loss of curve correction following 22 years of Boston brace treatment was 10° (SD ± 7.0) and that no Minimal Clinical Important Difference concerning the loss of correction between groups had previously been published, our statistical analysis showed that with a sufficient power of 0.8 and α value of 0.05, in order to see a difference of 5° (which is the approximate middle of the reported 10°) with 7° standard deviation between equivalent groups, at least 32 patients had to be enrolled in each of the two groups of the study.

Standard statistical methods have been used for descriptive statistics. Normally distributed continuous variables were analyzed by using an independent sample t test, non-normally distributed with the Mann–Whitney U test and categorical variables with chi square test. The normality of different groups’ data distribution was tested according to the “Kolmogorov-Smirnov” or the “Shapiro-Wilk” test. The hypothesis of equality of means was discarded when the probability of a type I error was 5 %. All statistical tests were two-tailed. Analyses were performed with the use of the SPSS statistical software (version 21, Chicago-IL).

## Results

### Demographics

Seventy seven patients (females: 71) with mean age 40.4 (±3.2) years were studied. The mean pre-brace scoliotic curve was 28.25 (±8.73) degrees and the mean Perdriolle score of the apical vertebra of the scoliotic deformity was 14.3 (±6.67). 30 patients had thoracic, 21 thoracolumbar, 8 lumbar and 18 double primary curve. 46 (59.7 %) of patients had pre brace Cobb angle lesser than 30^0^ and 31 (40.3 %) had greater than 30^0^. Mean time of the brace application was 2.71 (±1.29 years). They underwent long term follow up at a mean of 25.16 (±2.69) years after brace removal.

### Outcomes

#### Cohort results

In all 77 patients the mean scoliotic curve was reduced by 6.65 (±9.5) over the Short Term Follow-up; the curvature was increased however by 3.9 (±6.69) at the Long Term Follow-up. The mean pre brace Cobb angle of 28.25 (±8.73) was reduced to 21.58 (±11.54) following brace removal and increased 25.48 (±13.87) at Long Term Follow-up. There was however no significant difference in the mean Cobb angle of the cohort between pre brace and long term follow up period (*p* = 0.307). 55 patients (71.4 %) of the cohort completed the long term follow up with less than 30°, 14 patients (18.2 %) between 30–40° and 8 patients (10.4 %) with more than 40°. 5 (6.8 %) patients at Short Term Follow-up and 27 patients (35.1 %) at the Long Term Follow-up of the whole cohort demonstrated curve increase of more than 5^0^. 18 (39.1 %) of curves below 30^0^, 5 (22,7 %) of curves between 30 and 40^0^ and 4 (44,4 %) of curves greater than 40^0^ progressed more than 5^0^ at the long term follow up (*p* = 0.341).

#### Comparison between full time vs part time application

Thirty-five patients (45 %) had full time whereas 42 (55 %) part time application. The two groups were statistically comparable according to age, sex, employment and marital status, onset of menstruation, time of brace application and removal, duration of brace application, magnitude of the main scoliotic curve before bracing, rotation of the apical vertebra and the type of scoliotic curve (Tables 1 and 2).

**Table 1:** Comparison of socio-demographic data between part and full time braced patients, 25 years post bracing

		Mode of application	
		Part Time^a^	Full Time^a^
Age(years)^b^		40 (±3.1)	40.8 (±3.2)
Sex^c^	Male	3 (7.1)	3 (8.6)
	Female	39 (91.4)	32 (92.9)
Weight^b^		66.3 (±14.0)	63.8 (±12.7)
Height^b^		167 (±5.9)	166.2 (±6.7)
BMI^b^		23.7 (±4.9)	23.04 (±3.8)
Smoking^c^	no	15 (42.9)	19 (45.2)
	yes	20 (57.1)	23 (54.8)
Work status^c^	Sedentary	16 (45.7)	19(45.2)
	Part time standing	13 (37.1)	15 (35.7)
	Housekeeping	4 (11.4)	7 (16.7)
	Full time standing	2 (5.7)	1 (2.4)
Educational level^c^	Primary school	16 (45.7)	13 (31)
	High school	18 (51.4)	25 (59.5)
	University	1 (2.9)	4 (9.5)
Comorbidity^c^	no	14 (40.0)	23 (54.8)
	yes	21 (60.0)	19 (45.2)
Marital status^c^	Not married	11 (31.4)	10 (23.8)
	Married	30 (68.6)	24 (71.4)
Born children^c^	0–1	17 (53.1)	17 (43.6)
	2–3	15 (46.9)	20 (51.3)
	4–5	0 (0)	2 (5.1)

^a^No significant differences in the socio-demographic data of the patients were identified between the two treatment groups (Test performed using x^2^ test, student t-test or Mann–Whitney test)

^b^The values are given as the mean with the standard deviation in parentheses

^c^The values are given as raw numbers with the percentages in parentheses

**Table 2:** Comparative data between part and full time braced patients at baseline, short and long-term follow-up

		Mode of application		
		Part Time	Full Time	*p* value
Baseline characteristics at brace application				
Index age of first time application^a^		13.24 (±1.9)	13.19 (±1.49)	0.55^c^
Onset of menstruation^a^		12.52 (±0.95)	12.48(±1.17)	0.70^c^
Curve magnitude (Cobb angle/ out of brace)^a^		28.71 (±9.02)	27.86 (±8.58)	0.84^c^
Curve type^b^	Thoracic	12 (34.3)	18 (42.9)	0.38^d^
	Thoracolumbar	12 (34.3)	9 (21.4)	
	Lumbar	2 (5.7)	6 (14.3)	
	Double primary	9 (25.7)	9 (21.4)	
Rotation of the apical vertebra (Perdriolle score)^a^		15.09 (±6.9)	13.69 (±6.48)	0.40^c^
Curve magnitude (Cobb angle/ in-brace correction) after first brace application^a^		18.2 (±8.46)	16.55 (±9.9)	0.32^c^
Initial curve correction after brace application^a^		−10.17 (±7.36)	−11.31 (±5.8)	0.65^c^
Short Term Follow Up (post bracing)				
Age at brace removal^a^		16.07 (±1.09)	15.8 (±1.34)	0.40^c^
Duration of brace application^a^		2.82 (±1.59)	2.61 (±0.97)	0.95^c^
Curve magnitude (Cobb angle/ out of brace) at brace removal^a^		20.43 (±10.3)	22.55 (±13.03)	0.51^c^
Curve correction at brace removal compared with pre-brace angle^a^		−8.26 (±9.58)	−5.31 (±9.34)	0.09^c^
Long Term Follow Up ( 25 years post bracing)				
Time since brace removal^a^		24.95 (±2.45)	25.34 (±2.89)	0.10^c^
Curve magnitude (Cobb angle) at Long Term Follow Up^a^		24.37 (±12.4)	26.4 (±15.08)	0.58^c^
Type of main scoliotic curve^b^	Single thoracic	11 (31.4)	14 (33.3)	0.88^d^
	Double Thoracic	2 (5.7)	3 (7.1)	
	Double Major	8 (22.9)	12 (28.6)	
	Triple major	3 (8.6)	2 (4.8)	
	Thoracolumbar	8 (22.9)	6 (14.3)	
	Lumbar	3 (8.6)	5 (11.9)	
Rotation of the apical vertebra (Perdriolle score)^a^		13.66 (±9.03)	14.86 (±9.01)	0.52^c^
Loss of main curve correction from brace removal to Long Term Follow Up^a^		3.94 (±6.43)	3.86 (±6.98)	0.87^c^
Loss of correction > 5 degrees^b^		10 (28.6)	17 (40.5)	0.27^d^

^a^The values are given as the mean with the standard deviation in parentheses

^b^The values are given as raw numbers with the percentages in parentheses

^c^Test performed using Mann–Whitney test

^d^Test performed using x^2^ test

There was no statistical significant difference in the mean curve correction between the two groups at Short term follow-up (Table 2). Long term follow up revealed moderate increase in the Cobb angle in both groups. The mean Cobb angle increase was 3.86 (±6.98) in the Full Time and 3.94 (±6.43) degrees in the Part Time application group. Difference between groups was not statistically significant (*p* = 0.87). 17 patients in the full time group and 10 patients in the part time group demonstrated curve increase of more than 5^0^. This difference was not significant (*p* = 0.27) between the two groups (Table 2). At Long Term follow up there was no significant difference in the quality of life between the two groups (Table 3).

**Table 3:** Quality of life scores between partially and full time braced patients at long-term follow up

Questionnaire	Subscale	Mode of brace application		
		Part Time	Full Time	*P* Value
SF-36^a^	Physical functioning	80.0 (±16.9)	79.5 (±19.9)	0.78^b^
	Limitation due to physical	85.3 (±17.6)	83.9 (±22.0)	0.97^b^
	Limitation due to emotional	79,4 (±23.3)	83.9 (±20.9)	0,28^b^
	Energy	61.0 (±20.1)	62.2 (±22.8)	0.73^b^
	Emotional	64.4 (±21.5)	70.1 (±21.9)	0.19^b^
	Social functioning	81.6 (±22.2)	85.1 (±23.6)	0.31^b^
	Pain	80.2 (±18.7)	71.8 (±24.4)	0.18^b^
	General Health	66.3 (±17.6)	69.1 (±21.7)	0,37^b^
	Health change	52.9 (±19.2)	55.9 (±18.9)	0.52^b^
	Total	73.8 (±14.4)	74.9 (±17.4)	0.46^b^
Oswestry Disability Index	Pain Intensity	0.47 (±0.78)	0.57 (±0.96)	0.83^b^
	Personal Care	0.06 (±0.23)	0.12 (±0.32)	0.37^b^
	Lifting	1.5 (±0.82)	1.55 (±0.86)	0.97^b^
	Walking	0.94 (±0.88)	0.79 (±0.89)	0.42^b^
	Sitting	1.09 (±0.57)	1.14 (±0.56)	0.67^b^
	Standing	1.74 (±0.66)	1.57 (±0.73)	0.27^b^
	Sleeping	0.38 (±0.49)	0.36 (±0.53)	0.72^b^
	Sex Life	0.29 (±0.52)	0.24 (±0.48)	0.61^b^
	Social Life	0.47 (±0.82)	0.69 (±0.92)	0.24^b^
	Travelling	0.91 (±0.45)	0.93 (±0.74)	0.99^b^
	Total	15.7 (±8.72)	15.9 (±11.04)	0.64^b^

^a^The values are given as the mean with the standard deviation in parentheses

^b^Tests performed using Mann–Whitney test

## Discussion

Bracing remains the main conservative treatment in Adolescent Idiopathic Scoliosis [5, 6]. The selection of the appropriate patient and the time and duration of application are still controversial [14, 15].

Most of the long-term follow-up studies showed that successful bracing delays significantly the progression of the curve during its application [8, 16–18]. In addition it results in a moderate correction of the curve compared with the index curve, at the time of the brace removal. Whether the protective role of bracing remains active following brace removal is also unclear. A limited number of studies support the corrective effect of bracing at the time of the long term follow-up [8, 16–18].

Nachemson and Danielson [8] reported long term results of 109 patients at 22 years following Boston or Milwaukee brace discontinuation. The mean initial curve magnitude of 33.2 before bracing was lessened to 24.7 during the bracing time and subsequently increased up to 29.7 at brace removal. The mean loss of correction at 22 years after brace discontinuation was 7.9°. Lange et al. [17] confirmed similar long term results in 215 patients suffering from AIS or Juvenile Idiopathic Scoliosis at 25 years after Boston brace removal. The mean Cobb angle deterioration at the long term evaluation was 4.1° after brace removal. In our study, the mean prebrace Cobb angle of 28.2 (±8.7) was reduced during brace application to 17.3 (±9.2), increased slightly at the short time follow up to 21.5 (±11.8) and further increased at the 25 years follow up to 25.4 (±13.8). The mean pre brace Cobb angle however was not significantly different at 25 years follow-up, demonstrating stability of the sample. 55 patients (71.4 %) of the cohort completed the long term follow up with less than 30° Cobb angle. Outliers with curve progression following brace removal > 5° were observed in 27/77 (35 %) of our cohort. Our results coincide to the recorded experience in the published literature regarding the course of curve progression following brace removal. Bracing seems to be an effective treatment method with good long term results in appropriate patients.

The proper time of brace application however remains controversial [5, 19, 20]. In a prospective randomised study , Weinstein et al. reported that the benefit of brace application in Adolescent Idiopathic Scoliosis is correlated with longer daily time of bracing (*p* < 0.001) [5]. A meta-analysis by Rowe et al. concluded that 23 h bracing was significantly more successful than 8 or 16 h treatment ( *p* < 0.0001) [20].

Compliance however, remains the major concern for the full time bracing [21–23]. Compliance monitoring with heat sensors was reported to increase compliance [22, 23]. Part-time or night time bracing [24] has been introduced to address the poor compliance and psychological burden of full-time bracing; evidence however for their efficacy remains limited. Part time bracing has been reported to be as effective as full time bracing for smaller curves [16]. Allington et al. in a retrospective study reported that less time application was equally effective compared to full time (*p* < 0.18) for curves even greater than 30° [25].

In our study we found that 18 h bracing was equally effective compared to 23 h (*p* = 0.51) thus a reasonable compromise between compliance and effectiveness could be achieved. A limitation of our study and a crucial compromise however was that the division of the two groups was based only on the reported bracing time from patients. No other compliance certification method was used at the time of brace application.

The critical question arising however is the long term protective effect of the part time application. We ask this question 25 years following brace removal in a cohort of 77 patients treated with full or part time brace application for a mean of 3 years during their adolescence. We found no significant difference in both loss of correction and quality of life between groups (Tables 2 and 3).

Wiley raised the same hypothesis for a shorter however follow up period [16]. In his retrospective study, 50 Adolescent Idiopathic Scoliosis patients treated with Boston brace were evaluated at an average of 9.8 years after brace removal. Patients had curves of 35 to 45° and braced for a mean of 2.2 years. Patients were retrospectively divided in three groups; 18 or more hours, 12–18 and 0–12 h per day. The authors concluded that 18 h or more were sufficient for maintaining curve correction at a long term follow up [16].

## Conclusions

Our cohort demonstrated moderate loss of correction 25 years post successful bracing during adolescence. The mean cohort Cobb angle however remained stable 25 years post bracing. We found no difference in terms of long term results and progression between 18 and 23 h of self-reported bracing hours. In conclusion, bracing could be effective for long term in selected patients with Adolescent Idiopathic Scoliosis; since compliance was not objectively measured, we don’t feel confident to give any indication about everyday dosage.
